# Supplementary material for: Planned development and evaluation protocol of two versions of a web-based computer-tailored nutrition education intervention aimed at adults, including cognitive and environmental feedback
Source: BMC Public Health. 2014 Jan 17;14:47. doi: 10.1186/1471-2458-14-47 (PMC3915224; doi:10.1186/1471-2458-14-47)
Supplement: Additional file 1: Table S1 — Theoretical methods and applications used in the intervention. [file 1471-2458-14-47-S1.pdf]

## Table S1. Theoretical methods and applications used in the intervention

| Determinant | Theoretical method | Parameter for use | Practical application |
|---|---|---|---|
| <u>Phase I: Pre-action</u> | | | |
| *Knowledge* | Provide general information about behavior (IMB) [1] | | Providing facts and correct misperceptions about the target behaviors intake of fruit, vegetables, high-energy snacks and fat (e.g. 'what is the recommendation for fruit consumption?' / 'vegetables contain a lot of pesticides') |
| | Provide information on consequences of not complying with guideline of behavior (TRA, TPB, SCT, IMB) [1] | | Providing information about the possible health consequences of not complying to the dietary guidelines of each target behavior |
| *Awareness* | Monitoring behavior | | Filling out assessment questionnaires regarding the target behavior |
| | Information about personal risk (PAPM) [2] | Messages presented as individual, undeniable, on same dimension, congruent with actual risk, and cumulative rather than for one occasion; messages presented with qualitative and quantitative examples [2] | Providing tailored behavioral, normative and comparative feedback on intake of target behavior |
| *Attitude* | Belief selection (TPB) [2] | Requires investigation of the current beliefs of individual before choosing belief on which to intervene [2] | Selecting advantages and disadvantages regarding target behavior from a predefined list |

| | | | |
|---|---|---|---|
| | Persuasive communication (PCM, ELM, SCT) [2] [3] | Consideration of the source, message, channel and receiver [2], having knowledge of the assumptions in the target group, message should not deviate too much from initial ideas of target group [3] | Using persuasive communication in feedback on selected advantages and disadvantages to strengthen advantages and weaken disadvantages / correct misperceptions regarding the target behavior<br><br>Providing a ‘taste-test’ in which participants can state their preference regarding the taste of fruits and vegetables, followed by a list of products fitting preference |
| | Modeling (SCT) [2] | Target group can identify with the model, a coping model is provided, reinforcement of the model is visible [2, 4] | Peer stories, incorporated in feedback on disadvantages regarding target behavior |
| *Self-efficacy* | Prompting identification of barriers (SCT) [1] | Choosing barriers which are important to individuals | Selecting barriers regarding the target behavior from a predefined list<br><br>Selecting difficult situations regarding the target behavior from a predefined list |
| | Persuasive communication (PCM, ELM, SCT) [2, 3] | | Using persuasive communication in feedback on how to overcome selected barriers / difficult situations regarding the target behavior |
| | Provide instructions (SCT) [1] | | Providing information and tips on how to be able to perform the target behavior, |

| | | | |
|---|---|---|---|
| | | | incorporated in feedback on selected barriers / difficult situations |
| | Modeling (SCT) [1-3] | Target group can identify with the model, a coping model is provided, reinforcement of the model is visible [2, 4] | Peer stories regarding the target behavior, incorporated in feedback on selected barriers / difficult situations |
| | Prompt specific goal setting (CT) [1, 5]<br><br>Action planning (implementation intentions) [2, 6, 7] | Goals should be specific and challenging, but feasible [5]<br><br>Existing positive intention [2] | Setting goals regarding the target behavior in an open-ended format<br><br>Formulating action plans regarding the target behavior in an open-ended format |
| *Intention* | Prompt intention formation (TRA, TPB, SCT, IMB) [1] | | Stimulate participants to change target behavior when they are not complying with the recommendation (see 'awareness')<br><br>Changing negative attitude regarding target behavior to stimulate participants to change target behavior (see 'attitude') |
| *Availability and location of food products in home environment* | Monitoring the arrangement of food products in the home environment<br><br>Persuasive communication (PCM, ELM, SCT) [2, 3] | | Filling out a short assessment questionnaire about the arrangement of food products in the home environment regarding the target behavior<br><br>Using persuasive communication in feedback on availability and location of |

| | | | |
|---|---|---|---|
| | | | food products in home environment regarding the target behavior |
| | Provide instructions (SCT) [1] | | Providing suggestions to rearrange home environment into a more supportive environment regarding the target behavior |
| | Create a more supportive home environment | | Stimulating participants to rearrange home environment regarding the target behavior based on the provided instructions |
| *Perceived availability and price of products in local food environment* | Tailored objective information | | Providing information about objective availability and price of healthy food products regarding the target behavior in the supermarket the participant tends to go to |
| <u>Phase II: Action</u> | | | |
| *Awareness of progression of behavior change* | Monitoring progression of behavior change | | Stimulating participants to keep daily record of progress regarding (the change in) the target behavior |
| *Self-efficacy* | Prompting identification of barriers (SCT) [1] | | Keeping track of situations which caused failure to change target behavior |
| <u>Phase III: evaluation</u> | | | |
| *Awareness of progression of behavior change* | Monitoring progression of behavior change | | Filling out an assessment questionnaire about reaching goal (and intake) regarding |

| | | | |
|---|---|---|---|
| | | | the target behavior |
| | Provide feedback on performance (CT) [1, 8, 9] | | Providing tailored feedback on progress of behavior change regarding the target behavior (e.g. whether someone did or did not eat more fruit) |
| | Prompt self-monitoring of behavior (CT) [1] | | Stimulating participants to keep record of their target behavior and to act on it when diverging from goal |
| | Prompt review of behavioral goals (CT) [1] | | Stimulating participants to review achievement of goal and, if applicable, adapt goal |
| *Attitude* | Decisional balance | | Participants specify which advantages / disadvantages they perceive for changing the target behavior<br><br>Participants review their decisional balance |
| *Self-efficacy* | Coping planning [7] | Identification of high risk situations and practicing coping response | Identifying situations which caused failure to change target behavior and making plans to avoid or manage these situations the next time (i.e. formulating coping plans in an open-ended format) |
| | Goal setting (CT) [1, 5] | Goals should be specific and challenging, but feasible [5] | Participants can adapt their goal regarding the same target behavior (i.e. lower or increase the goal)<br><br>Participants can set a goal |

| | | | |
|---|---|---|---|
| | | | for another target behavior (i.e. participants are redirected to another target behavior) |
| | Provide instructions (SCT) [1] | | Providing information and tips on how participants can maintain their new behavior on the long-term |

**IMB** = Information-Motivation-Behavioral Skills Model, **TRA** = Theory of Reasoned Action, **TPB** = Theory of Planned Behavior, **SCT** = Social Cognitive Theory, **PAPM** = Precaution Adoption Process Model, **PCM** = Persuasion Communication Matrix, **ELM** = Elaboration Likelihood Model, **CT** = Control Theory

## References


1. Abraham C, Michie S: **A taxonomy of behavior change techniques used in interventions.** *Health Psychol* 2008, **27:**379-387.
2. Bartholomew LK, Parcel GS, Kok G, Gottlieb NH, Fernández ME: *Planning Health Promotion Programs: An Intervention Mapping Approach.* San Francisco: Jossey-Bass; 2011.
3. Kok G, Lechner L, Meertens R, Brug J: **Theorieën en methodieken van verandering (Theories and methodologies of change).** In *Gezondheidsvoorlichting en Gedragsverandering: Een Planmatige Aanpak (Health Education and Behavioral Change: A Systematic Approach). Volume* 8. Edited by Brug J, van Assema P, Lechner L. Assen: Van Gorcum; 2012
4. Kok G, van Empelen P: **Interventie ontwikkeling (Intervention development).** In *Gezondheidsvoorlichting en Gedragsverandering: Een Planmatige Aanpak (Health Education and Behavioral Change: A Systematic Approach).* Edited by Brug J, van Assema P, Lechner L. Assen: Van Gorcum; 2012: 125-144
5. Strecher VJ, Seijts GH, Kok GJ, Latham GP, Glasgow R, DeVellis B, Meertens RM, Bulger DW: **Goal setting as a strategy for health behavior change** *Health Educ Behav* 1995, **22:**190-200.
6. Gollwitzer PM, Sheeran P: **Implementation intentions and goal achievement: a meta-analysis of effects and processes.** *Advances in Experimental Social Psychology* 2006, **38:**69-119.
7. Sniehotta FF, Schwarzer R, Scholz U, Schüz B: **Action planning and coping planning for long-term lifestyle change: theory and assessment.** *Eur J Soc Psychol* 2005, **35:**565-576.
8. Mento AJ, Steel RP, Karren RJ: **A meta-analytic study of the effects of goal setting on task performance: 1966–1984.** *Organ behav hum decis process* 1987, **39:**52-83.

9. Neubert MJ: **The value of feedback and goal setting over goal setting alone and potential moderators of this effect: a meta-analysis.** *Human Performance* 1998, **11:**321-335.
